# FunctSNP: an R package to link SNPs to functional knowledge and dbAutoMaker: a suite of Perl scripts to build SNP databases

**DOI:** 10.1186/1471-2105-11-311

**Published:** 2010-06-09

**Authors:** Stephen J Goodswen, Cedric Gondro, Nathan S Watson-Haigh, Haja N Kadarmideen

**Affiliations:** 1 CSIRO Livestock Industries, Davies Laboratory, University Drive, Townsville, QLD 4810, Australia; 2 Centre for Genetic Analysis and Applications, University of New England, Armidale, NSW 2351, Australia

## Abstract

**Background:**

Whole genome association studies using highly dense single nucleotide polymorphisms (SNPs) are a set of methods to identify DNA markers associated with variation in a particular complex trait of interest. One of the main outcomes from these studies is a subset of statistically significant SNPs. Finding the potential biological functions of such SNPs can be an important step towards further use in human and agricultural populations (e.g., for identifying genes related to susceptibility to complex diseases or genes playing key roles in development or performance). The current challenge is that the information holding the clues to SNP functions is distributed across many different databases. Efficient bioinformatics tools are therefore needed to seamlessly integrate up-to-date functional information on SNPs. Many web services have arisen to meet the challenge but most work only within the framework of human medical research. Although we acknowledge the importance of human research, we identify there is a need for SNP annotation tools for other organisms.

**Description:**

We introduce an R package called FunctSNP, which is the user interface to custom built species-specific databases. The local relational databases contain SNP data together with functional annotations extracted from online resources. FunctSNP provides a unified bioinformatics resource to link SNPs with functional knowledge (e.g., genes, pathways, ontologies). We also introduce dbAutoMaker, a suite of Perl scripts, which can be scheduled to run periodically to automatically create/update the customised SNP databases. We illustrate the use of FunctSNP with a livestock example, but the approach and software tools presented here can be applied also to human and other organisms.

**Conclusions:**

Finding the potential functional significance of SNPs is important when further using the outcomes from whole genome association studies. FunctSNP is unique in that it is the only R package that links SNPs to functional annotation. FunctSNP interfaces to local SNP customised databases which can be built for any species contained in the National Center for Biotechnology Information dbSNP database.

## Background

Whole genome association studies (WGAS) are a set of methods to study common genetic variation across the entire genome of organisms with the purpose of identifying associations between genetic polymorphisms with observable phenotypic variation in traits. Single nucleotide polymorphisms (SNPs) are the most common genetic variant. There are essentially two advances that have allowed WGAS. Firstly, the huge increase in the availability of SNPs. For example, the dbSNP database housed by the National Center for Biotechnology Information (NCBI) contains millions of SNPs for many model species. Secondly, the falling cost and rapid improvements in SNP genotyping technology. One of the main outcomes from WGAS is a subset of SNPs at a specified level of statistical significance (p - values) and false discovery rates (FDR). These SNPs are currently used in two conventional, albeit alternative, approaches depending on the requirements of the researcher: 1) a molecular biologist usually searches for candidate genes in the region of the significant SNP and then does functional experiments, which could include targeted sequencing; 2) a geneticist uses significant SNPs as DNA markers for predicting disease risks, phenotypic performance or breeding value in individuals and may use this information in associated trait selection (marker-assisted selection/gene-assisted selection).

One of the major challenges for a researcher using WGAS is minimising false positive rates while maintaining the power to identify true positive associations [1]. WGAS relies on linkage disequilibrium (LD) between DNA markers and causal variants. SNPs located near each other on a chromosome are said to be in LD if they tend to be inherited together more often than can be expected by chance [1]. Unlike the SNP chip design for humans that had input from the HapMap project to assist in its SNP selection, the tag SNP selection for most other species is not strictly based on LD knowledge. For many species the LD patterns are still to be conclusively determined and therefore the tag SNPs for the genotyping SNP chip are selected to be evenly distributed [2].

In WGAS, association does not imply causation. The significant SNPs derived from WGAS can be generally classified into 4 types: 1) a causal SNP that contributes to variation in the complex trait; 2) a SNP with no known biological effect but in LD to an untagged SNP (not genotyped) that contributes to variation in the complex trait; 3) a SNP with an association only - no *known *biological effect or linkage to a causal SNP; 4) a SNP *not *associated with the complex trait (a false positive). Since the type of significant SNP following WGAS is not distinguishable to the researcher, there is a potential to perform functional experiments based on a false-positive SNP and/or use a SNP as a marker that is neither the causal variant nor in LD with the causal variant.

Currently, the only approach to determine if a WGAS derived significant SNP is a true positive and in LD with the causal variant is to replicate the association findings in an independent population of adequate size [1]. Hence, a need exists for SNP annotation tools that can assist a researcher in distinguishing the type of significant SNP. The tool would need to first collect and integrate all existing biological functional information from online resources. Then provide an interface to access, analyse, and present the information so that the researcher may make informed judgments as to whether a SNP could be a causal variant (type 1) or is in LD with a causal variant (type 2).

Table 1 shows a list of the main current bioinformatics tools for SNP annotation. The tools are web-based and predominantly work within the framework of human medical research. In May 2009, dbSNP held genetic polymorphisms for 45 organisms and 15 of the organisms have sequenced genomes. We have developed a unified bioinformatics tool, called FunctSNP, to address the need to provide SNP annotation for other species as well as humans. FunctSNP is an R package which provides a set of functions to query species-specific local relational databases within the widely used statistical programming language R http://www.r-project.org/.

**Table 1:** Existing SNP annotation tools

Name	Description	Type^+^	Species	External Resources Used
SNPit [15]	Analyses the potential functional significance of SNPs derived from genome wide association studies.	W	Human	dbSNP, EntrezGene, UCSC Browser, HGMD, ECR Browser, Haplotter, SIFT
SNP Function Portal [16]	Explores the functional implication of SNP alleles	WD	Human	dbSNP, UniSTS, NCBI ideogram, Entrez Gene, NCBI human genome assembly, HapMap II project
Pupasuite [17]	SNP prioritization and characterisation	WD	Human Mouse Rat	Ensembl, Gene Ontology, OMIM
SNPnexus [18]	Provides functional annotation for both novel and public SNPs	WD	Human	NCBI RefSeq, UCSC Known Genes, VEGA, AceView
LS-SNP/PDB [19]	Annotates non-synonymous SNPs mapped to Protein Data Bank structures	WD	Human	UniProtKB, Genome Browser, dbSNP, PDB
F-SNP [20]	Computationally predicts functional SNPs for disease association studies	WD	Human	PolyPhen, SIFT, SNPeffect, SNPs3D, LS-SNP, ESEfinder, RescueESE, ESRSearch, PESX, Ensembl, TFSearch, Consite, GoldenPath, Ensembl, KinasePhos, OGPET, Sulfinator, GoldenPath
FANS [21]	Functional analysis of novel SNPs	WD	Human Mouse	NCBI, Ensembl, UCSC BLAT, Rescue-ESE Fas-ESS, SIFT
SNPer [22]	Facilitates the retrieval and use of Human SNPs for high-throughput research purposes.	WD	Human	dbSNP, Goldenpath, LocusLink, GeneOntology, SWISS-PROT, OMIM, Unigene
SNAP [23]	An integrated SNP annotation platform	W	Human	Ensembl, UCSC, Uniprot, UniProt, Pfam, DAS-CBS, MINT, BIND, KEGG, TreeFam
SNPeffect [13]	Predicts the effect of non-synonymous SNPs on the molecular phenotype of proteins.	W	Human	Ensembl human databases
SNPs3D [24]	Assigns molecular functional effects of non-synonymous SNPs based on structure and sequence analysis.	W	Human	dbSNP, SWIIS-Prot, RefSeq, Bioscience, Gene Ontology, KEGG, BIND, OMIM, HGMD, Entrez Gene

^+^Type = Application type.

W = Web interface to external resources: WD = Web interface to custom database.

For many of the bioinformatics tools, third party applications are required for upstream and downstream research analyses, which in most cases require data format changes. The R environment has a global data currency of vectors and data frames so there are few data conversion concerns between R packages. For instance, there are two R packages called GenABEL [3] and SNPassoc [4], which perform genome wide association studies. FunctSNP ideally complements these R packages by linking their output to SNP annotation. In addition, there are R packages called GSA [5], GoSim [6], GeneNet [7], and snp.plotter [8] for further downstream analysis to provide advanced algorithms, and coloured graphs.

**Figure 1:**
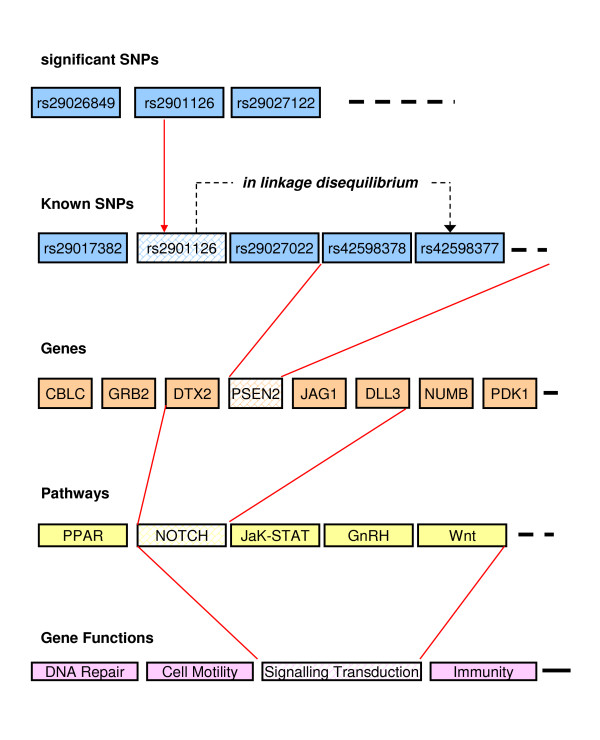
**Schematic to show the process of finding the potential function of significant SNPs**. rs2901126 is a supposed significant SNP but is not located on a gene and has no known biological effect (as is the case for most WGAS derived significant SNPs). Is it a false positive? In this example, rs2901126 is in linkage disequilibrium to an untagged SNP (a SNP not on the genotyping array). The untagged SNP *can *be linked to gene functions that may provide an indication it contributes to variation in a complex trait. Therefore, rs2901126 is potentially a reliable DNA marker.

A primary goal of FunctSNP is to help screen and select the WGAS significant SNPs which are more likely to be reliable as DNA markers. WGAS makes the direct association between SNP variation and phenotype variation. Whereas in reality SNP variation affects phenotype variation through the effects it has on multiple intervening biological pathways and networks [9]. FunctSNP aims to link WGAS derived SNPs to these biological pathways and gene networks. The FunctSNP functions within R allow screening of SNPs by: 1) determining their physical location with respect to genes. As a general rule, the closer a significant SNP is to the potential causal variant the more likely they will be in LD; 2) finding evidence for a functional role of the significant SNP. It is well known that over 90% of significant SNPs are not located on genes. Therefore, if the significant SNP is not located on a gene, FunctSNP functions can search in close proximity to the significant SNP for a known SNP (not genotyped) that is on a gene, within a biological pathway, and with a biological function known to be associated with the trait of interest [10]. Figure 1 shows an example, through a schematic diagram, of the process of finding the potential function of significant SNPs.

## Construction and content

The biological information required for SNP annotation is publically available in online databases. Although most of these online resources provide cross-links to other databases, it is not straightforward to extract the relevant research data in one query. Furthermore, even though the databases contain well-annotated data, finding and managing the data of interest among the large assortment of data can be a challenge. Our solution to superfluous and disseminated data was to link SNPs with functional knowledge within species-specific local relational databases.

### Database creation

**Figure 2:**
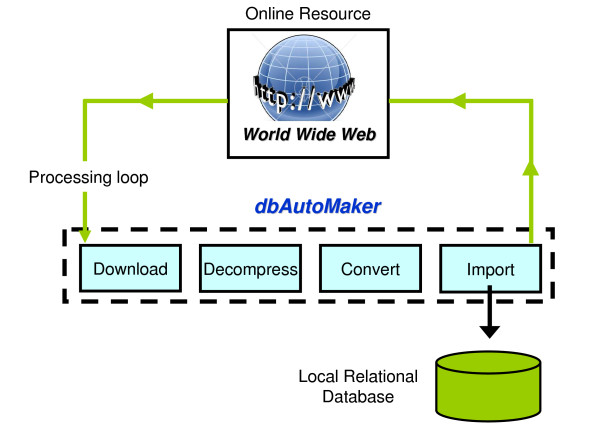
**Schematic to show the dbAutoMaker database creation process**. dbAutoMaker is a suite of Perl scripts developed to automatically repeat the steps of 'download-decompress-convert-import' to create a local database with data extracted from any number of resources.

We have made available 5 species-specific databases for cattle, chicken, sheep, pig, and human. Each database contains the most pertinent SNP related data extracted from 7 online resources (Table 2). We use the public domain relational database management system (RDMS) SQLite http://www.sqlite.org/. It stores data in a file that is platform independent, provides batch mode functionality, and is the preferred RDMS for R. The data contained in the online resources is dynamic, so we have developed a suite of Perl scripts, collectively called dbAutoMaker, to provide a framework in which to automate the process of creating a local database with data extracted from any number of resources. Figure 2 shows the 4 automated steps performed by dbAutoMaker: 1) download - reads, from a configuration file, a list of Uniform Resource Locators (URLs) associated with the online resource data files and then downloads them; 2) decompress - automatically decompresses files with suffixes tar, tgz, gz, and zip; 3) convert - converts the data into an SQL format that can be imported to a SQLite database. There is currently no standardized interchange format for transporting data from one database to the next. The data formats encountered were Microsoft SQL Server, MySQL, XML, and ASCII text file (flat file). Generic Perl scripts that work in conjunction with configuration files were developed to handle each data format; 4) import - imports the data into the SQLite database using SQLite batch mode command files created by the Perl scripts in step 3. These 4 steps are repeated for each online resource. dbAutoMaker can be scheduled to run periodically to ensure the local database is always up-to-date. No further intervention is required once the framework and schedule is setup.

**Table 2:** External resources used to create SNP customised database

Acronym	Name	Link	Resource
dbSNP	Single Nucleotide Polymorphism database	http://www.ncbi.nlm.nih.gov/	SNPs
GO	Gene Ontology	http://www.geneontology.org/	Genes and gene product attributes
KEGG	Kyoto Encyclopaedia of Genes and Genomes	http://www.genome.jp/kegg/	Biological pathways
UniProt	Universal Protein Resource	http://www.uniprot.org/	Protein sequences and functional information
QTLdb^++^	Animal Quantitative Trait Locus database	http://www.animalgenome.org/QTLdb/	Quantitative Trait Loci data (QTL)
OMIA^++^	Online Mendelian Inheritance in Animals	http://omia.angis.org.au/	Genes, inherited disorders and traits
HomoloGene		http://www.ncbi.nlm.nih.gov/homologene	Homolog detection

^++^Not applicable to *Homo sapiens*

The core of dbAutoMaker is a species-specific configuration file in an INI file format. Although we have demonstrated dbAutoMaker using mainly livestock species, it can be configured by the user to create a SNP database for any species contained in NCBI's dbSNP [for more detail refer to dbAutoMaker User Guide on http://www.csiro.au/science/dbAutoMaker.html].

**Figure 3:**
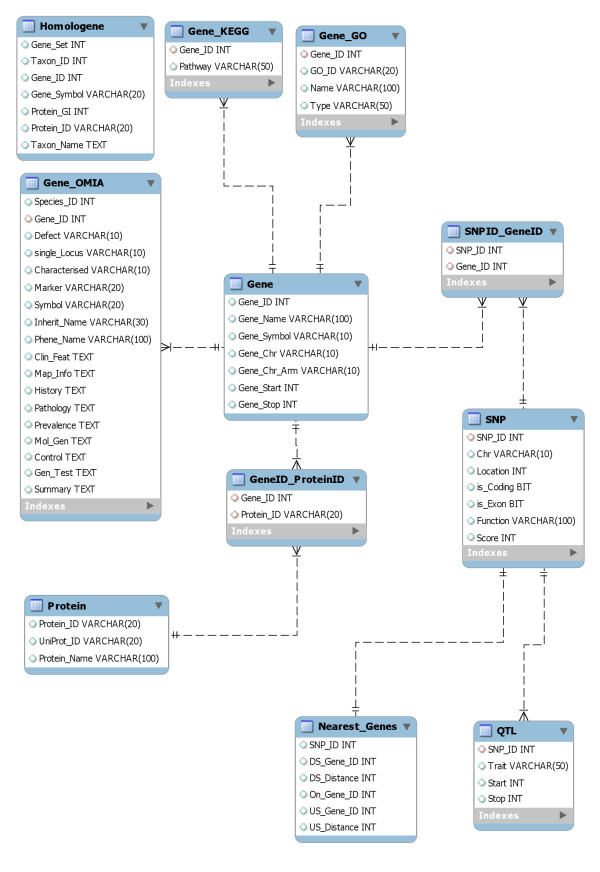
**Database Schema for custom built SNP database**.

Figure 3 shows the schema for the SNP relational database. Each table can be linked through the SNP_ID or Gene_ID attributes and consequently all data is integrated.

The SQLite databases can be downloaded from http://www.csiro.au/science/FunctSNP.html. The user can directly query the database using Structured Query Language (SQL) through the SQLite program. However, we recommend FunctSNP as the database user interface.

The quality of results that can be expected from FunctSNP is reliant on the amount and reliability of information extracted from the online resources. For example, based on the amount of data available, the *Homo sapiens *database is information rich in comparison to all other species. *Bos taurus *and *Gallus gallus *databases are poor in comparison to *Homo sapiens *but substantially richer compared to *Sus scrofa *and *Ovis aries*. The table in Additional file 1 is a summary of the number of entries in the FunctSNP species-specific databases that were extracted from the online resources. Given time it is expected that the amount of available data for all species will dramatically increase.

## Utility

FunctSNP provides its utility through R functions. So far, 20 R functions (Table 3) have been implemented for analysing the biological information. Although FunctSNP is primarily designed to link WGAS derived significant SNPs to biological information, it is flexible and there is no restriction on how, or what order, the R functions can be used. The FunctSNP functions are described in full in the manual pages supplied with the R package, so are only briefly mentioned here.

**Table 3:** FunctSNP R Functions

Name	Description
addSpecies	Adds a new species to the list of species recognized by FunctSNP
downloadDB	Download pre-assembled species-specific databases
getGeneID	Extract gene ID information using SNP IDs or SNP locations
getGenes	Extract gene information using SNP IDs or Gene IDs
getGenesByDist	Extract gene ID within a specified distance from a SNP
getGO	Extract gene ontology using SNP IDs or Gene IDs
getHighScoreSNP	Extract highest scoring SNP using SNP IDs or SNP locations
getHomolo	Extract homologous genes using SNP IDs or Gene IDs
getKEGG	Extract pathway names using SNP IDs or Gene IDs
getNearGenes	Find nearest genes to either SNP IDs or SNP locations
getOMIA	Extract OMIA using SNP IDs or Gene IDs
getProteins	Extract protein information using SNP IDs or Gene IDs
getSNPID	Extract SNP ID using Gene IDs or SNP locations
getSNPs	Extract SNP information using SNP IDs or Gene IDs
getTraits	Extract traits associated with QTL regions using SNP IDs or Gene IDs
installedDBs	Displays the local available databases
makeDB	Build a species-specific database from external sources
setSpecies	Sets the default species
supportedSpecies	Displays the supported species
userAddedSpecies	Displays the species added by user

The FunctSNP functions provide access to the biological information contained within species-specific databases. Information such as: SNP chromosomal location, exon/intron status, synonymous/non-synonymous effect, SNPs in Quantitative Trait Loci (QTL) regions, biological pathways, GO terms, and protein products for related genes. Multiple databases (one for each species) can be queried in the same R session.

Central to the SNP databases are the SNP ID (NCBI's dbSNP rs# cluster ID) and gene ID (NCBI's gene ID). Consequently, the majority of FunctSNP functions accept, as input, a vector of either SNP IDs or gene IDs by specifying what type of IDs are being passed to the function using id.type. For example:

snps <- getSNPs(ids,id.type = "snp")

The getSNPs () function returns SNP information (e.g., chromosome number, chromosomal location, protein coding status etc.) for each SNP ID in the ids vector.

snps <- getSNPs(ids,id.type = "gene")

This time, the getSNPs () function returns SNP information for *all *SNPs residing within each of the genes specified by their gene IDs in the ids vector.

As a general guideline to the user in prioritising the SNPs most likely to have significant functional roles, all SNPs in the database have been scored. The total score is comprised of a simple sum of two components. The first component is a score according to the SNP location and the type of amino acid alteration it may cause. We use NCBI's dbSNP function classification for SNPs on genes. The table in Additional file 2 shows the function classification in the first column and the FunctSNP score value in the second column. The classification is ranked and scored according to the likelihood of the function type altering the amino acid. For example, a SNP is given a maximum score if the polymorphism alters an amino acid. We acknowledge that scoring is subjective and therefore it is our intention in a future version of FunctSNP to allow a user-defined score value for each function classification. The second component used in the calculation of the total score is a cumulative score according to the amount of supporting biological information found in the database. If the SNP can be linked to proteins, GO terms, KEGG pathways, QTL regions, and homologous genes, the score is incremented for each linkage. For example, a SNP assigned a score of 26 (the maximum possible score) is derived as follows: 20 (SNP alters codon to make an altered amino acid) + 1 (SNP is located in a QTL region) + 1 (Protein information found) + 1 (one or more GO terms found) + 1 (one or more KEGG pathways found) + 1 (one or more OMIA information found) + 1 (homologous genes found).

There are R functions to: find the nearest genes to each SNP ID entered, using a chromosomal base pairs search distance - getGenesByDist(); find the nearest high scoring SNP to each SNP ID, or chromosomal location entered - getHighScoreSNP(); find homologous genes across all species or a specified species for each SNP ID, or gene ID entered - getHomolo(); find the quantitative trait loci (QTL) region associated to a trait (as annotated within QTLdb database) for each SNP ID or gene ID entered - getTraits(); and extract SNP ID for each chromosomal location entered - getSNPID(). There are also R functions to download the latest customised SNP database for a particular species (currently, databases for cattle, chicken, sheep, pig, and human are available) - downloadDB(); and a function to invoke dbAutoMaker for the user to make their own databases - makeDB().

### Program testing

To test FunctSNP, we used 10 k SNP genotype data from a yet to be published livestock WGAS. From this 10 k SNP data, 165 SNPs had been identified as being significantly associated with a particular trait of economic interest. These SNPs were sufficiently quality controlled and accepted as reliable prior to using FunctSNP.

The aim of the test was to rank the 165 SNPs according to their likelihood of being reliable DNA markers. Our premise for reliable DNA markers is that WGAS derived SNPs should be either the causal variant or close to a SNP that is the causal variant. In FunctSNP, whether a SNP is a potential causal variant is determined by its assigned functional role and its links to biological information (e.g., pathways and gene ontology). The SNPs in the database have been scored in accordance with this premise. Although, high scoring SNPs in FunctSNP are the most likely to have significant functional roles, the expertise of the researcher is required to make that important mental link between the biological information provided and its relevance to the trait of interest. We also use the general rule that the closer a WGAS derived SNP is to a high scoring SNP the more likely the SNPs will be in LD. However, the researcher needs to be aware that this rule does not always hold true as there are blocks of SNPs along the chromosome with higher and lower degrees of LD. For example, for the most part the closer 2 SNPs are together the stronger the LD is going to be, nevertheless, it is possible that the 2 SNPs are located on different blocks of LD.

The test involved 4 different runs based on the following criteria that determined the data input to the FunctSNP functions: 1) using all 165 WGAS derived significant SNPs; 2) using only significant SNPs with a score greater than 7; 3) using SNPs with a score greater than 11 from the database that are within 10,000 base pairs from the 165 significant SNPs; and 4) using SNPs from the database with a score greater than 24 that are located on genes within 10,000 base pairs from the 165 significant SNPs. The threshold score used is at the discretion of the user. SNPs with a score equal or greater than 20 are considered highly likely to have significant functional roles. The base pairs search distance is also subjective. Knowledge of the LD patterns for the research species will need to govern the distance used.

Table 4 is a summary of the amount of information obtained from each test criterion. In run 1, all 165 of the significant SNPs were input into the functions getGenes(), getProteins(), getKEGG(), getGO(), and getHomolo (). Although, the SNPs are located on 52 genes which can be linked to 23 proteins, 43 biological pathways, and 259 GO terms only 1 significant SNP could be considered a candidate causal variant. We did not analyse the output from this run any further and proceeded with test run 2. In order to prune the output information this run used significant SNPs with a score greater than 7 as input to functions. The GO IDs, KEGG pathway names, and UniProt IDs that were output from run 2, 3 and 4 were used as input to their associated websites to find additional clues (a step that we recommend in the FunctSNP demonstration guide located at http://www.csiro.au/science/FunctSNP.html). For example, GO IDs e.g., GO: 0001875, can be input under "Search the Gene Ontology Database" at http://www.geneontology.org/; KEGG pathway names e.g., path:bta04620, input "bta" under "Select Prefix" and "04620" under "Enter Keywords" at http://www.genome.jp/kegg/pathway.html; and UniProt IDs e.g., Q4GZT3, input under UniProt homepage at http://www.uniprot.org/.

**Table 4:** Shows a summary of the output count after 4 program test runs

	Number of SNPs ...			Number of entries found in database for SNPs meeting input criteria ^++^				
**#**	**meeting input criteria^$$^**	**derived from WGAS meeting criteria**	**found in database with score = > 20**	**Genes**	**Proteins**	**Pathways**	**Gene Ontology**	**Homologous Genes(with Homo sapiens)**

1	165	165	1	52	23	43	259	47
2	7	7	1	7	4	19	29	5
3	15	1	8	15	9	29	114	13
4	11	0	11	6	10	21	126	6

# = test run number^$$ ^criteria determines the number of SNPs input into program

1: all 165 significant SNPs

2: significant SNPs with score greater than 7

3: SNPs with score greater than 11 within 10,000 base pairs from significant SNPs

4: SNP with score greater than 24 located on genes within 10,000 base pairs from significant SNPs.

^++ ^Example: For run 3, the highest scoring SNPs within 10,000 base pairs from 165 significant SNPs were found in database; 15 of the found SNPs met the criteria of score > 11; 1 out of the 15 SNPs found was a WGAS derived SNP; 8 SNPs from the 15 have a score = > 20. 15 SNPs are located on 15 genes with 9 protein products. The genes are involved in 29 pathways, have 114 Gene Ontology terms, and are homologous with 13 human genes.

The purpose of run 3 was to find the highest scoring SNPs in the database residing in close proximity to the significant SNPs. We found 15 SNPs located on 15 different genes and 8 of these SNPs were considered to have significant functional roles. Similarly, the purpose of run 4 was to find genes in close proximity to the significant SNPs and then find the highest scoring SNPs residing on these genes. We found 6 genes in close proximity that contained 11 SNPs with significant functional roles. Based on the results of the 4 tests we were able to rank the 165 SNPs according to their likelihood of being reliable DNA markers and concluded that the top 8 were realistic candidate DNA markers. In particular, 2 WGAS derived SNPs were identified to be in close proximity to 2 genes influencing biological pathways that literature have linked to the WGAS trait of interest. See additional file 3 for FunctSNP commands used in testing program.

## Discussion

As part of the ongoing development, we will monitor web resources for new and improved sources of data that can be incorporated into FunctSNP. The design of dbAutoMaker allows data to be extracted from new online resources with minimal modification to dbAutoMaker's configuration file.

FunctSNP and our approach for identifying SNP markers currently make the assumption that all causal SNPs are located on genes. There are now many suggestions that non coding SNPs (e.g., SNPs in transcription factor binding sites, splice sites, repressor, promoter regions, and eQTLs [9]) could significantly contribute to variation in complex traits. Therefore, as part of the ongoing development, online resources (such as coliSNP [11], TRANSFAC [12], SNPeffect [13], eQTL Explorer [14]) are being investigated to find data for the functional role of non-coding SNPs with the intention of incorporating this data in FunctSNP databases.

## Conclusions

To take full advantage of WGAS, we need to make that essential link between the outcomes from WGAS and the information that exists about the function of genes and pathways. Whilst there are many emerging bioinformatics tools to make this link, we have developed a tool called FunctSNP which we believe is unique and addresses some of the shortcomings of existing tools. Firstly, FunctSNP is the only R package designed specifically for SNP annotation. There are considerable advantages over a web interface in integrating FunctSNP within the R statistical computing environment offering high quality graphics and data visualization tools. Operating in the same R environment, FunctSNP has notable complementary upstream and downstream research R packages. Secondly, the purpose built database for FunctSNP can include information about SNPs in other organisms besides human. We have developed an application called dbAutoMaker for a researcher to automatically build their own SNP database for any species contained in NCBI's dbSNP. We have scheduled dbAutoMaker to build databases for cattle, chicken, pig, sheep, and human on a monthly basis. These databases can easily be downloaded whenever required directly from the web or using a FunctSNP function.

We conclude that FunctSNP is a post-WGAS tool that provides an opportunity to screen and select for SNPs that have a higher likelihood to be related to variation in a particular complex trait of interest.

## Availability and requirements

The FunctSNP R package is available from CRAN:

http://cran.r-project.org/package=FunctSNP. In FunctSNP there are no new classes, no overloading, and default storage modes for R objects are used.

The software for dbAutoMaker is implemented in Perl and supported on Linux and MS Windows. Databases and source code are freely available from: http://www.csiro.au/science/FunctSNP.html and http://www.csiro.au/science/dbAutoMaker.html

## Authors' contributions

HNK conceived this systems genetics project, and developed and obtained the CSIRO research grant for the project. SJG, CG and NSW developed FunctSNP. SJG developed dbAutoMaker. CG and HNK supervised the project. All 4 authors wrote the paper, read and approved the final manuscript.

## Supplementary Material

Additional file 1 **Table - Summary of the number of entries in the customised species-specific SNP databases**. Click here for file

Additional file 2 **Table - FunctSNP SNP prioritisation**. Click here for file

Additional file 3 **FunctSNP commands used in testing program**. Click here for file
