# Supplementary material for: FunctSNP: an R package to link SNPs to functional knowledge and dbAutoMaker: a suite of Perl scripts to build SNP databases
Source: BMC Bioinformatics. 2010 Jun 9;11:311. doi: 10.1186/1471-2105-11-311 (PMC2901372; doi:10.1186/1471-2105-11-311)
Supplement: Additional file 1 — Table - Summary of the number of entries in the customised species-specific SNP databases. [file 1471-2105-11-311-S1.PDF]

Additional file 1 – Table – Summary of the number of entries in the customised species-specific SNP databases

[Data downloaded and table compiled in December 2009]

	Source of downloaded data							
	NCBI dbSNP		NCBI Gene	KEGG	GO	QTLdb	OMIA	Homologene
	Types of entry							
Species	SNPs	Functional SNPs ++	Genes ^^	Pathways	Terms	QTLs	Phenes **	Genes $$
Homo sapiens	14,708,752	143,679	40,591	14,164	123,942	--	--	19,571
Gallus gallus	3,293,383	16,134	19,936	6,991	45,286	1,863	179	13,803
Bos taurus	2,223,033	6,982	28,885	13,391	63,771	2,359	379	19,803
Sus scrofa	8,427	0	10,355	5,943	0	5,621	222	0
Ovis aries	0	0	1,190	0	0	84	188	0

++ Coding non-synonymous (nonsense, missense, frame shift)
^^ The number of genes includes confirmed genes and genes predicted by annotation processes
** A phene is information on the occurrence and inheritance of disorders and other familial traits
$$ The number of genes in the species genome that are placed in a homology group
-- Not applicable to *Homo sapiens*
