# Supplementary material for: FunctSNP: an R package to link SNPs to functional knowledge and dbAutoMaker: a suite of Perl scripts to build SNP databases
Source: BMC Bioinformatics. 2010 Jun 9;11:311. doi: 10.1186/1471-2105-11-311 (PMC2901372; doi:10.1186/1471-2105-11-311)
Supplement: Additional file 2 — Table - FunctSNP SNP prioritisation. [file 1471-2105-11-311-S2.PDF]

Additional file 2 – Table – FunctSNP SNP prioritisation

NCBI's Function Classification	Score
Changes to STOP codon (nonsense)	20
Alters codon to make an altered amino acid (missense)	20
indel SNP causing frame shift	20
Synonymous amino acid change	15
Within 3' 0.5kb to a gene	10
Within 5' 2kb to a gene	10
Untranslated region	5
Protein coding: synonymy unknown	5
3 prime untranslated	5
5 prime untranslated region	5
Slice site - 3 prime acceptor dinucleotide	5
Slice site - 5 prime donor dinucleotide	5
Intron	1
