# Supplementary material for: FunctSNP: an R package to link SNPs to functional knowledge and dbAutoMaker: a suite of Perl scripts to build SNP databases
Source: BMC Bioinformatics. 2010 Jun 9;11:311. doi: 10.1186/1471-2105-11-311 (PMC2901372; doi:10.1186/1471-2105-11-311)
Supplement: Additional file 3 — FunctSNP commands used in testing program. [file 1471-2105-11-311-S3.PDF]

```
##################################################
#  FunctSNP Commands for case study test
##################################################

library(FunctSNP)

downloadDB ("bta")

setSpecies("bta")

snp_ids <- read.table ("CRC Sig_SNPs.dat",header=FALSE)

# ========================================
#  Run 1 - Use all WGAS significant SNPs
# ========================================

gene_ids <- getGeneIDs(snp_ids,snpid.keep=FALSE,loc.keep=FALSE)

gene_ids <- getGenes (snp_ids)

snps <- getSNPs (snp_ids)

snps_on_gene <- gene_ids[,c("SNP_ID")]

traits <- getTraits (snps_on_gene)

go_terms <- getGO (snps_on_gene)

pathways <- getKEGG (snps_on_gene)

proteins <- getProteins (snps_on_gene)

taxons <- c (9606)

homolo <-getHomolo (snps_on_gene,id.type="snp",taxon.ids=taxons)

# ====================================================
#  Run 2 - significant SNPs with score greater than 7
# =====================================================

hs_snps <- getSNPs (snps_on_gene)

row.idx <- which(hs_snps [,"Score"]> 7)

hs_snps <- hs_snps [row.idx,c("SNP_ID")]

hs_genes <- getGenes (hs_snps)

hs_traits <- getTraits (hs_snps)

hs_go_terms <- getGO (hs_snps)

hs_pathways <- getKEGG (hs_snps)

hs_proteins <- getProteins (hs_snps)

hs_snp_info <- getSNPs (hs_snps)

homolo <-getHomolo (hs_snps,id.type="snp",taxon.ids="9606")
```

```r
# ===================================================================
#  Run 3 - SNPs with score greater than 11 within 10,000 base pairs
#  from significant SNPs
# =================================================================

snps_with_hs <- getHighScoreSNP(snp_ids,dist=10000)

row.idx <- which(snps_with_hs [,"Score"]> 11)

snps_with_hs <- snps_with_hs [row.idx,c("SNP_ID")]

snps_with_hs_info <- getSNPs (snps_with_hs)

nr_genes <- getGenes (snps_with_hs)

nr_traits <- getTraits (snps_with_hs)

nr_go_terms <- getGO (snps_with_hs)

nr_pathways <- getKEGG (snps_with_hs)

nr_proteins <- getProteins (snps_with_hs)

nr_omia <- getOMIA (snps_with_hs)

nr_snp_info <- getSNPs (snps_with_hs)

homolo <-getHomolo (snps_with_hs,id.type="snp",taxon.ids="9606")

# ==================================================================
#  Run 4 - SNP with score greater than 24 located on genes within
#  10,000 base pairs from significant SNPs.
# ================================================================

ng_genes <- getGenesByDist (snp_ids,dist=10000)

ng_snps <- getSNPs (ng_genes,"gene")

row.idx <- which(ng_snps [,"Score"]> 24)

ng_snps <- ng_snps [row.idx,c("SNP_ID")]

ng_snps_info <- getSNPs (ng_snps)

ng_gene_IDs <- getGeneID (ng_snps,loc.keep=FALSE,snpid.keep=FALSE)

ng_genes <- getGenes (ng_gene_IDs,"gene")

ng_genes <- getGenes (ng_snps)

ng_traits <- getTraits (ng_snps)

ng_go_terms <- getGO (ng_snps)

ng_pathways <- getKEGG (ng_snps)

ng_proteins <- getProteins (ng_snps)

ng_omia <- getOMIA (ng_snps)

homolo <-getHomolo (ng_snps,id.type="snp",taxon.ids="9606")
```
